# Ambient Sound-Based Collaborative Localization of Indeterministic Devices

**DOI:** 10.3390/s16091478

**Published:** 2016-09-14

**Authors:** Jacob Kamminga, Duc Le, Paul Havinga

**Affiliations:** 1 Pervasive Systems Group, University of Twente, Enschede 7522 NB, The Netherlands; v.d.le@utwente.nl (D.L.); p.j.m.havinga@utwente.nl (P.H.); 2 ASTRON, Dwingeloo 7991 PD, The Netherlands

**Keywords:** sound localization, collaborative localization, opportunistic localization, wireless sensor networks, smartphones, Android, input latency

## Abstract

Localization is essential in wireless sensor networks. To our knowledge, no prior work has utilized low-cost devices for collaborative localization based on only ambient sound, without the support of local infrastructure. The reason may be the fact that most low-cost devices are indeterministic and suffer from uncertain input latencies. This uncertainty makes accurate localization challenging. Therefore, we present a collaborative localization algorithm (Cooperative Localization on Android with ambient Sound Sources (CLASS)) that simultaneously localizes the position of indeterministic devices and ambient sound sources without local infrastructure. The CLASS algorithm deals with the uncertainty by splitting the devices into subsets so that outliers can be removed from the time difference of arrival values and localization results. Since Android is indeterministic, we select Android devices to evaluate our approach. The algorithm is evaluated with an outdoor experiment and achieves a mean Root Mean Square Error (RMSE) of 2.18 m with a standard deviation of 0.22 m. Estimated directions towards the sound sources have a mean RMSE of 17.5° and a standard deviation of 2.3°. These results show that it is feasible to simultaneously achieve a relative positioning of both devices and sound sources with sufficient accuracy, even when using non-deterministic devices and platforms, such as Android.

## 1. Introduction

Wireless Sensor Networks (WSNs) and opportunistic sensing are significant technologies that have been researched intensively over recent years [1,2]. Simple low-cost sensors, networked through wireless links and deployed in large numbers, enable a large amount of applications for monitoring and controlling homes, cities and the environment. Networked sensors are used in numerous applications, such as military target tracking and surveillance, natural disaster relief, health monitoring, hazardous environment exploration and seismic sensing [1]. Opportunistic sensing exploits the opportunistic communication between groups of mobile devices, such as smartphones, to share each other’s content, resources and services [3].

**Figure 1:**
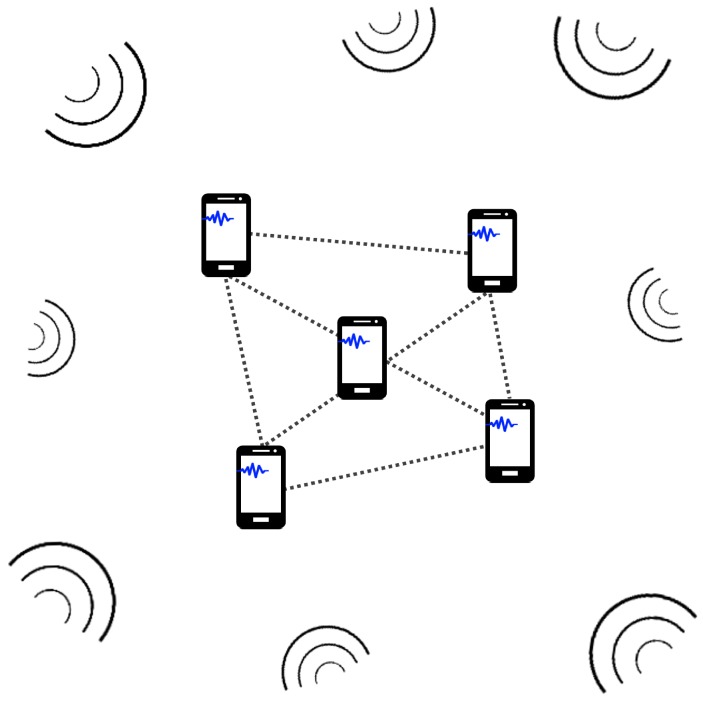
A group of devices that are able to communicate with each other can exploit ambient sounds to localize themselves and the origins of the sounds.

The capability of self-localization is highly desired in WSNs and opportunistic sensing applications [4]. Acquired measurement data are often meaningless when the location where they were measured is unknown. Location information can be used for coverage guaranties, deployment optimization, data routing, location services and target tracking. Typically, each device would know its own position, for example from the Global Positioning System (GPS). However, GPS devices require much energy, are relatively expensive, add size to a sensor node and might suffer from limited connectivity, such as in an indoor environment, in rugged terrains or dense cities and forests. These constraints require the investigation of techniques other than GPS [5]. Many localization techniques require the use of existing local infrastructure, such as anchor nodes with known positions. Remote WSNs and disaster scenarios, where infrastructure might not be present or malfunctioning, require localization techniques that do not rely on local infrastructure. In these cases, communication between wireless nodes can be used to improve the accuracy of location information in WSNs [6]. Techniques that accomplish this are known as collaborative localization, cooperative localization and network localization [7]. Numerous methods have been researched to preform cooperative localization [5]. One of these methods is based on Time Of Arrival (TOA) measurements. A signal is transmitted by an anchor node, and each device records the time when it receives the respective signal. Knowing the speed of the signal, this difference in time relates directly to the distance between the two devices and the anchor node. This method requires highly accurate clock synchronization between the devices and the anchor node. A variation of this method is based on Time Difference Of Arrival (TDOA) measurements. TDOA estimation requires the measurements of the difference in time between the signals arriving at two devices. The advantage that TDOA has over TOA is that the devices performing the measurements do no not have to synchronize their clocks with the device that produces the signal. This opens up the opportunity to exploit signals that are already present in the environment. Ambient sound signals are ubiquitous and can be exploited to preform TDOA-based cooperative localization. Additionally, it is possible to simultaneously localize the events that produced these sound signals while self-localization is preformed, without the use of existing infrastructure [8,9,10,11]. In disastrous scenarios, such as an explosion or gun shots in a crowd, these sound events can be simultaneously localized. Figure 1 denotes a group of devices that can communicate with each other and concurrently process sounds from the environment.

To the best of our knowledge, no prior work has exploited indeterministic devices for collaborative localization based on only ambient sound, without the support of local infrastructure. The uncertainty introduced by indeterminism and uncertain input latencies makes accurate localization challenging. Since the Android Operating System (OS) runs on numerous low-cost devices in the worldwide market and is an indeterministic OS, we select Android devices to evaluate our approach to collaborative localization with only ambient sound. In this paper, we present the Cooperative Localization on Android with ambient Sound Sources (CLASS) algorithm. The CLASS algorithm simultaneously localizes the position of indeterministic devices and ambient sound without requiring local infrastructure. The CLASS algorithm deals with the uncertainty by splitting the devices into subsets so that outliers can be removed from the time difference of arrival values and localization results. Besides the use on smartphones, our approach can also be used on simple sensor nodes in a Wireless Sensor Network (WSN).

Accurate timing properties are desirable for collaborative localization because a small error in time quickly relates to a large error in space (10 ms in TDOA relates to 3.4 m). One of the key characteristics of a real-time OS is the consistency in the amount of time the OS requires to complete a task. The variability in the completion times is denoted as jitter. Android is not a real-time OS, which makes the task completion times vary greatly and cannot be guaranteed. Therefore, it is a daunting challenge to perform collaborative localization with Android devices. In this paper, we investigate the limitations of implementing the Android OS for collaborative localization. We found that many parts of the current Android architecture contribute to inaccurate measurements; thus, improving the localization accuracy would require a completely new architecture and more accurate hardware. Previous work researched the ability to change the Android architecture in order to add real-time features [12,13]. This approach is hard to apply on different types of hardware and, therefore, not universal. Our approach allows applications to run on inexpensive hardware that is available in abundance, as opposed to complex, expensive high-end systems. When Android improves in all aspects needed, our approach is still beneficial, as even then, the accuracy will be improved.

### 1.1. Challenges

Localization by sound on low-cost devices introduces multiple challenges, such as:

- Setting up an ad hoc network for sound localization
- Time synchronization amongst devices in the network
- Detecting and identifying sound events that can be used for localization
- Audio latency and jitter in the hardware platform and operating system
- Dealing with inaccurate measurements
- Localizing devices and sound event origins

These challenges and related issues will be discussed in the respective sections of this paper.

### 1.2. Paper Contributions and Organization

The contributions of this paper are as follows:

- To the best of our knowledge, this work is the first to exploit indeterministic devices for collaborative localization based on only ambient sound, without the support of local infrastructure
- We investigate Android’s indeterministic behavior and applicability for collaborative localization. This provides an insight into the requirements of collaborative localization and the limitations of indeterministic devices.
- We present the CLASS algorithm that takes indeterministic behavior into account and preforms collaborative localization on smartphones.
- Our approach can be applied when errors are introduced by utilizing different types of phones or inexpensive, simpler hardware platforms.
- We assess the performance of the CLASS algorithm on an outdoor testbed of Android devices.

The remainder of this paper is organized as follows: Section 2 discusses prior work on the subject. In Section 3, we formulate the problem of localizing a set of devices with a set of directions toward sound events while not knowing any information regarding their locations. The CLASS algorithm that can apply this technique on Android devices is introduced in Section 4. Our Android application and an outdoor experiment are discussed and evaluated in Section 5. Finally, the paper is concluded in Section 6.

## 2. Related Work

Localizing an emitter or receiver in a WSN has been studied extensively over recent years [4,5]. In the field of localization, using only acoustic signals, two approaches can be distinguished; localization with and without anchor devices.

### 2.1. Target Localization Utilizing Anchor Devices with Known Positions

Localizing a node when the base stations are known has been studied by many. Harter et al. introduce a platform that enables applications to follow mobile users and/or objects inside a building with so-called “bat” nodes [14]. After overhearing its unique identifier, which is emitted by a base node, a bat node emits an ultrasound signal that is picked up by an array of receivers mounted in the ceiling. The location of the bat node is obtained through multilateration with a few centimeters of accuracy. Although the system is accurate, it requires much infrastructure and is an obtrusive technology to the user.

Simon et al. introduce PinPtr, an ad hoc wireless sensor network system that detects and accurately locates shooters [15]. PinPtr detects and measures the TOA of muzzle blasts and shock waves from a gunshot. Measurements are routed to a base station that computes the location of the gunshot. The hardware was synchronized with an error that stayed below 17.2 µs during a four-hour experiment. The error in time synchronization between Android devices will be much higher than this [16]. PinPtr requires a ranging phase in order to determine the relative positions of the motes. Ranging is a phase in the localization algorithm where devices estimate the respective distances between them and others. This is a drawback because the motes require a sounding device that has a limited range of approximately 10 m.

Shang et al. use Android devices as anchors at known locations to estimate the sound source location [16]. The devices themselves are not located and are assumed to accurately know their location. The authors find the TDOA by means of cross correlation between sound recordings that were recorded at different known locations. The recorded sound events and corresponding time stamps are centrally processed. The TDOA value contains the synchronization offset and start time error. These two errors are estimated and subtracted from the time delay to obtain the calibrated TDOA for the positioning of the source. The target localization errors of their acoustics-based method are within ±15 cm in the x-direction and ±80 cm in the y-direction. The authors found that multiple independent location estimates can be averaged to yield much better localization accuracy. The measurements were preformed in a quiet environment with low noise levels; outdoors, this method has not been tested and could possibly fail. The authors do not mention the inevitable offset in the anchor devices’ location, which will probably increase the error in the estimation of the target’s location.

### 2.2. Collaborative Localization Utilizing Only Sound Signals

The complexity of the localization problem increases when base stations with known positions are not present. Most research for this approach also localizes the location and time of emission of the sound event since this information is inherent in the measurement data.

Hennecke et al. [17] focus on the calibration of low-quality unsynchronized mobile phones. The authors rely on acoustic calibration signals and show that ranging can be used to compensate for poor time synchronization between devices. However, approaches that require ranging are not well suited for outdoor applications, simply because a smart phone will not be able to emit a distinguishable sound event over larger distances. Therefore, it is better to look at approaches that require no ranging.

Thrun et al. are some of the first authors to introduce a solution towards localization without any other information than TDOA measurements of sound events between multiple devices [8,9]. In their approach, the authors rely on the Far Field Approximation (FFA). In order to simplify the localization problem, most prior works assume that sound events originate (infinitely) far away. The FFA is refined by Kuang et al. [18], and their experimental validation gives a strong indication that a FFA is a feasible approach for getting direct estimates, as well as initial estimates for other solvers.

Wendeberg et al. [10,11] have successfully localized a group of mobile devices without the need for any further infrastructure besides ambient sound and a Wi-Fi network. They devised a TDOA method to localize a network of eight Apple laptops and iPhones with a positioning accuracy of approximately 1 m. The Apple products they used contain a High Precision Event Timer (HPET); thus, it becomes possible to synchronize the devices quite accurately. iPhones are known to have a much better audio pipeline and do not suffer from input latencies that much. Wendeberg et al. were able to synchronize the devices within an accuracy of 0.1 ms through the Network Time Protocol (NTP). We found that in Android, this accuracy in time synchronization, even utilizing the NTP, is currently not possible, and the measurements are very noisy. When another WSN synchronization protocol is used on a non-dedicated hardware platform, such as Android devices, the inaccuracies introduced by time synchronization are expected to be magnitudes larger. Therefore, it is important to research mechanisms that can utilize TDOA measurements with large errors. In order to deal with noisy data, Burgess et al. present an algorithm that uses the Random Sample Consensus (RANSAC) paradigm [19]. Their method simultaneously solves the calibration problem and removes severe outliers, which is a common problem in TOA applications. With two indoor environment experiments, using dedicated hardware with real-time features that was highly synchronized in time, their work achieved a Root Mean Square Error (RMSE) of 2.35 cm and 3.95 cm on microphones and speakers for their respective positions. Our approach presented in this paper focuses on implementing a similar technique that is evaluated on Android devices, with real measurements. We investigate where inaccuracies originate in Android and assess the performance of a RANSAC-inspired mechanism with noisy measurement data that was acquired on an outdoor testbed.

## 3. Problem Formulation

In this paper, we address the problem of localizing a set *X* of *M* mobile devices by utilizing a set *A* of *N* sound events. All devices in the set do not have any information regarding their location, but are able to communicate with each other and are poorly synchronized in time. For the ease of representation, the sensors and sound sources are located in a 2D plane, but the technique can easily be generalized to use 3D positions. We assume that the detected sound waves travel from the source to the devices in a straight line. In order to simplify the localization problem, we rely on the Far Field Approximation (FFA) [18]. The FFA assumes that sound events originate from (infinitely) far away. Under this assumption, a sound wave from sound event *j* arrives at each device at the same incident angle $\alpha_{j}$. In other words, the sound waves connecting the location of a sound event $\left(a_{j},b_{j}\right)$ with each of the devices $\left(x_{i},y_{i}\right)$ are approximately parallel for all *i* (but not for all *j*) [9,20]. Now, we do not need to find the relation between all devices and a sound event, but merely one direction towards it. Therefore, we assume that all sound events in *A* originate from an unknown location outside and around the constellation of devices (Figure 1). When the FFA does not hold, for example when a sound originates from near the device constellation, this could result in a large localization error. Burgess et al. found that the FFA gives good results as long as the distances between a sound event and device are four-times larger than inter-device distances [20]. Sound sources that originate from near the constellation of devices will increase the inaccuracy of the location results. However, the approach that we describe in this paper filters out inaccurate measurements, including sound sources that do not adhere to the FFA assumption. This filtering is discussed in Section 4.

The Time Of Arrival (TOA) of each sound event, at each device, is represented by a M×N TOA matrix *T*. In this matrix, the arrival time of sound event *j* at device *i* is denoted as $t_{i,j}$.

(1) $$T=\left(\begin{array}{cccc} t_{1,1} & t_{1,2} & \cdots & t_{1,M}\\ t_{2,1} & t_{2,2} & \cdots & t_{2,M}\\ \vdots & \vdots & \ddots & \vdots \\ t_{N,1} & t_{N,2} & \cdots & t_{N,M} \end{array}\right)$$

It is assumed that each sound event is well distinguished, e.g., in time or frequency, so that there is no data association problem. Sound event detection is discussed in Section 5.1.2. Each sound event represents one column in *T*. When no absolute positions are known, we can only look at the relative positions of the nodes. Therefore, we utilize the inter-device Time Difference Of Arrival (TDOA). The TDOA between Device 1 and all other devices can be denoted as:

(2) $$\Delta =\left(\begin{array}{cccc} t_{2,1}-t_{1,1} & t_{2,2}-t_{1,2} & \cdots & t_{2,M}-t_{1,M}\\ t_{3,1}-t_{1,1} & t_{3,2}-t_{1,2} & \cdots & t_{3,M}-t_{1,M}\\ \vdots & \vdots & \ddots & \vdots \\ t_{N,1}-t_{1,1} & t_{N,2}-t_{1,2} & \cdots & t_{N,M}-t_{1,M} \end{array}\right)$$

When we define the TDOA values not only relative to Node 1, but between all pair of nodes, we obtain the matrix Δ with dimensions M−1×N×M. In this matrix, the TDOA between node *i* and *k* for sound event *j* is denoted as $\Delta_{ijk}$. As discussed later in this paper, each TDOA measurement will contain a significant amount of noise due to inaccurate measurements. $\Delta_{ijk}$ therefore consists of the ground truth TDOA between device *i* and *k*, $\bar{\Delta_{ijk}}$, and the measurement noise *σ*:

(3) $$\Delta_{ijk}=\bar{\Delta_{ijk}}+\sigma$$

Because we know the speed of sound, we can relate the distance between two devices as follows:

(4) $$\begin{array}{cc} \Delta_{ijk}\cdot c & ={\left||X_{i}-A_{j}|\right|}_{2}-{\left||X_{k}-A_{j}|\right|}_{2} \end{array}$$

where *c* is the speed of sound and ${\left||X_{i}-A_{j}|\right|}_{2}$ the Euclidean distance between device *i* and sound event *j*. Because we rely on the FFA, the relation between the inter-device distance, sound event location and the respective TDOA value can be described as a function of the incident angle $\alpha_{j}$:

(5) $$\left(\begin{array}{cc} cos(\alpha_{j}) & sin(\alpha_{j}) \end{array}\right)\cdot \left(\begin{array}{c} x_{i}-x_{k}\\ y_{i}-y_{k} \end{array}\right)=\Delta_{ijk}\cdot c$$

The relation in Equation (5) can be used to form a least squares definition:

(6) $$J\left(X,\vec{\alpha }\right)=\sum_{\begin{array}{c} i=1\ldots N-1\\ j=1\ldots M\\ k=i+1\ldots N \end{array}}{\left(\left(\begin{array}{cc} cos(\alpha_{j}) & sin(\alpha_{j}) \end{array}\right)\cdot \left(\begin{array}{c} x_{i}-x_{k}\\ y_{i}-y_{k} \end{array}\right)-\Delta_{ijk}\cdot c\right)}^{2}$$

The device locations *X* and and angular directions $\vec{\alpha }$ can now be recovered by minimizing Equation (6) as follows:

(7) $$<X,\vec{\alpha }>=\underset{X,\vec{\alpha }}{\mathrm{argmin}}J\left(X,\vec{\alpha }\right)$$

## 4. A Collaborative Localization Algorithm: CLASS

**Figure 2:**
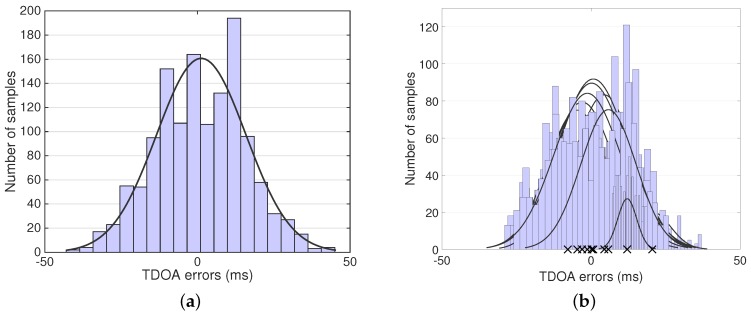
(**a**) Normal distribution of TDOA measurement errors; (**b**) Distributions of the subset TDOA measurement errors. The mean value of each sub-distribution is denoted by ×.

In this section, we describe the Cooperative Localization on Android with ambient Sound Sources (CLASS) algorithm. This algorithm localizes mobile devices based on ambient sound events at unknown locations. The algorithm outputs the relative position of the devices and the angular direction towards the origin of the sound events. In order to explain the intuition of our approach, we will first look at the quality of TDOA measurements on Android devices. We collected TOA measurements by generating sounds directly above 16 devices placed at an identical location. We recorded TOA values with the application described in Section 5.1. From these data, we can calculate the TDOA values through Equation (2) for all pairs of devices. All TDOA values should be close to zero, since all of the devices were positioned at an identical location. Figure 2a denotes the TDOA measurement error and indicates that it is normally distributed. This implies that averaging multiple TDOA measurements for sound events at an identical location can improve the accuracy of the result. Figure 2b denotes TDOA measurement error distributions for ten random subsets of devices. The figure shows that the mean, denoted by ×, for each subset varies significantly. This implies that the localization result will vary significantly each time a subset of different combinations of devices is used. Localizing a random subset of devices over multiple iterations will generate various location results for each device. We can weed out the outliers in the resulting set and yield a more accurate result.

**Figure 3:**
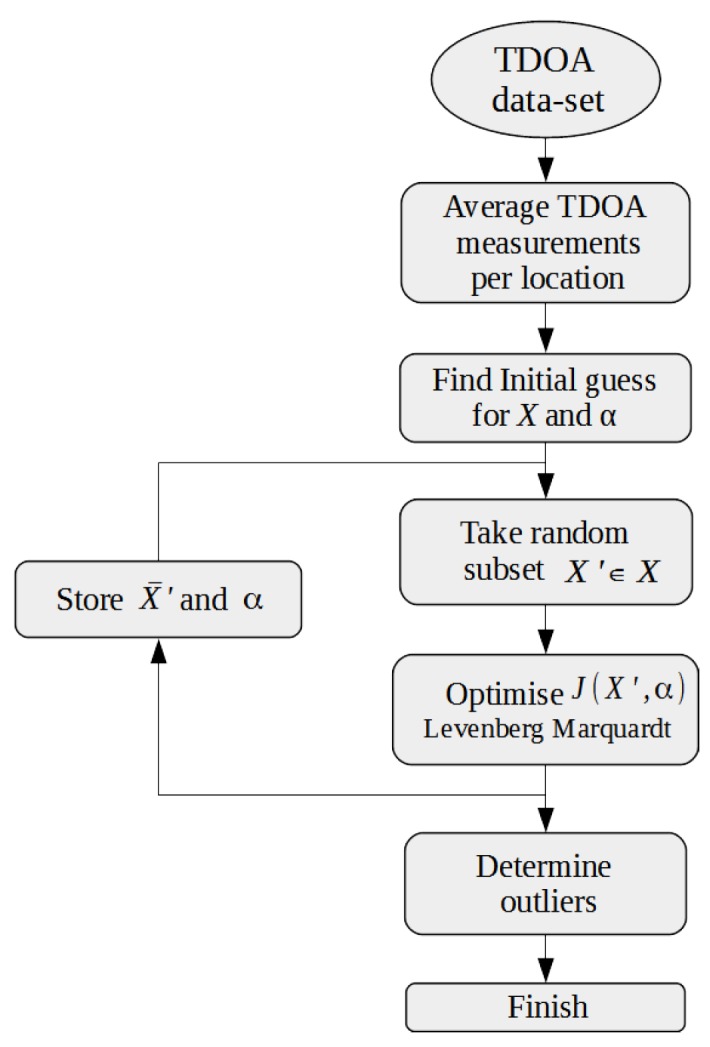
The Cooperative Localization on Android with ambient Sound Sources (CLASS) algorithm.

Figure 3 denotes the outline of the CLASS algorithm. The algorithm first averages TDOA values from an identical location that are inliers. The filtered TDOA data are then used to obtain an initial value for all device locations *X* and directions *α* through a minimal solver. The main loop of the algorithm then draws a random subset of devices $X^{\prime }$ and optimizes Equation (6) for *l* iterations. The location and direction solutions for each iteration are stored, and finally, we weed out outliers by means of a Histogram-Based Outlier Score (HBOS) algorithm. We will now discuss each part of the algorithm in the following sections.

### 4.1. Averaging TDOA Values for Events at Identical Locations

When it is possible to obtain multiple samples from one location by, e.g., splitting longer sounds over multiple events, a higher accuracy can be achieved [16]. The errors in TDOA measurements for an identical sound location are normally distributed (Figure 2a). Therefore, we can use the mean of TDOA values to determine which measurements are inliers. Algorithm 1 denotes how erroneous TDOA measurements are filtered out. We describe each subsequent step of the algorithm below.

**Algorithm 1:** TDOA filtering and averaging.
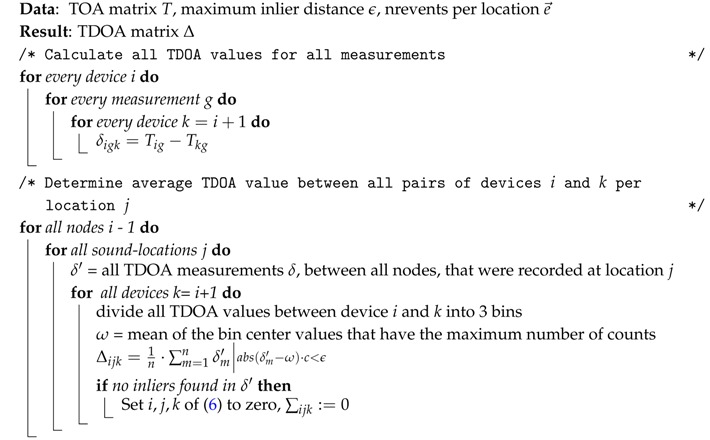


In the experimental dataset, four sound events have been recorded at each sound event location. We can use the averaged value of inliers for the respective value of $\Delta_{ijk}$. Outliers are extracted by means of a Histogram-Based Outlier Score (HBOS) algorithm that is described in Section 4.2. A value $\Delta_{ijk}$ is perceived as an inlier when the following condition holds:

(8) $$T_{ik}\cdot c<\epsilon$$

where $T_{ik}$ is the time difference of arrival between device *i* and *k*, *c* is the speed of sound and *ϵ* the maximum distance from the mean TDOA value *ω*, which is determined by the HBOS algorithm. The resulting set with inliers is averaged to form a more accurate value $\Delta_{ijk}$. When no inliers are found for a particular value $\Delta_{ijk}$, the information between device *i* and *k* for sound event *j* will not be taken into account when optimizing Equation (6). In other words, an erroneous measurement will not take part in the localization algorithm by setting the term for this combination of i,j,k of Equation (6) to zero, $\sum_{ijk}:=0$. Both devices still have relations with other devices that are more stable, and these can be used to determine their respective locations. Thus, not taking into account a TDOA value between a pair of nodes for one sound event location is not a problem. The method that filters and averages the TDOA values is presented as Algorithm 1. The first step of Algorithm 1 calculates the TDOA values between all pairs of devices for all sound events. We then use a HBOS (Algorithm 2) to select TDOA measurements that are inliers for each sound event between all pairs of devices. The TDOA values that are inliers are averaged and form a new TDOA value between each pair of devices for each location.

### 4.2. Histogram-Based Outlier Detection

An HBOS [21] algorithm is implemented to detect outliers in the TDOA measurements and device locations (x,y). Outliers in the localization results are caused by technological limitations of the hardware platform, synchronization errors and violation of the Far Field Approximation (FFA) due to sound sources that originate from near the device constellation. We chose a HBOS algorithm because it allows us to find outliers in a large dataset without prior training. The algorithm is presented in pseudocode as Algorithm 2, and we will discuss each step below.

**Algorithm 2:** HBOS filter.
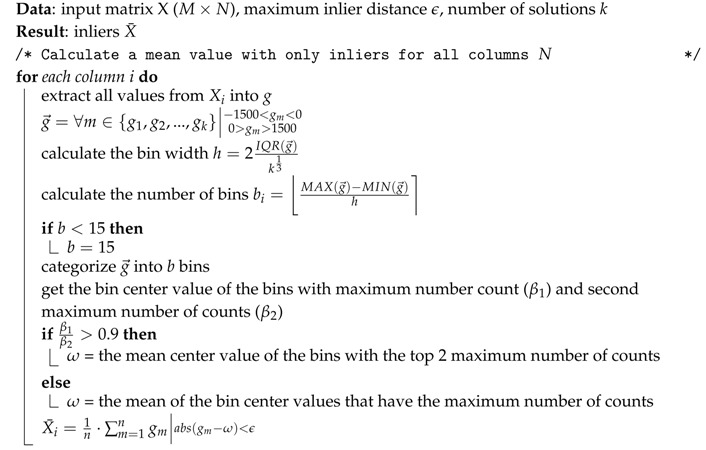


In order to determine which values in the set are inliers, a mean, upper and lower bound are first determined. The HBOS algorithm categorizes all values within a certain range into bins. This is done by creating a histogram of the vector $\vec{g}$. This vector represents either TDOA measurements for a single sound event origin or multiple location values for device *i*, ($x_{i}$, $y_{i}$). $\vec{g}$ has size 1×k. The bin-width *h* of the histogram is determined with the Freedman–Diaconis rule [22] as follows:

(9) $$h=2\frac{IQR(\vec{g})}{k^{\frac{1}{3}}}$$

where $IQR(\vec{g})$ is the interquartile range of the location data and *k* equals the number of observations in the set $\vec{g}$. The number of bins *b* is based on the size of the bins and the minimum and maximum value in $\vec{g}$:

(10) $$b=\left\lfloor \frac{MAX\left(\vec{g}\right)-MIN\left(\vec{g}\right)}{h}\right\rceil$$

where ⌊⌉ is the nearest integer. The minimum number of bins *b* is set to 15; fewer bins will not provide enough resolution for outlier detection. $\vec{g}$ can contain extremely severe outliers, which influence the determination of *b* negatively; $\vec{g}$ is therefore limited to:

(11) $$\vec{g}=\forall m\in \left\{g_{1},g_{2},...,g_{k}\right\}|\begin{array}{c} -1500<g_{m}<0\\ 0>g_{m}>1500 \end{array}$$

**Figure 4:**
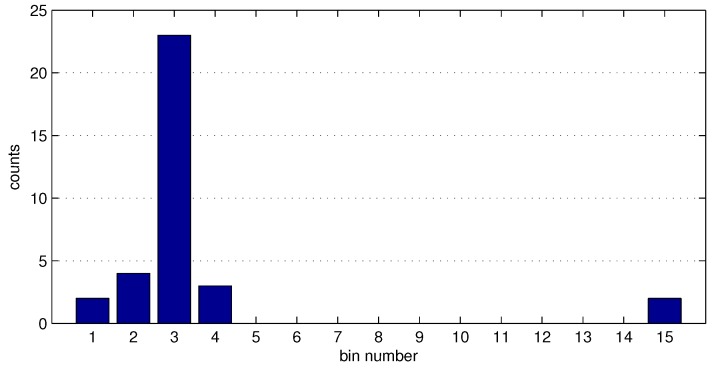
Bin classification and bin counts.

Figure 4 displays an example bar plot from a histogram of $\vec{g}$ where the amount of iterations k=35. In this example, most of the values in $\vec{g}$ are categorized in Bin 3, the center value that belongs to this bin is used as the mean value *ω* for inlier determination.

**Figure 5:**
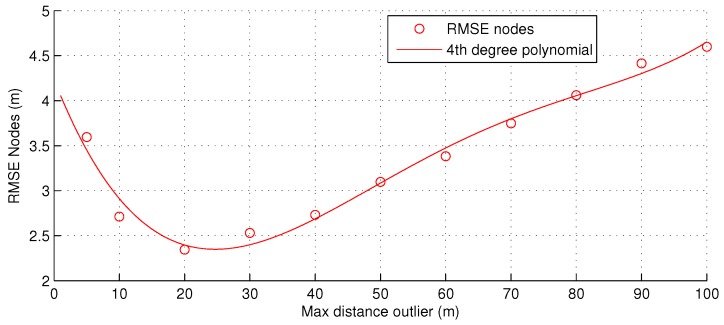
Varying the size of maximum distance *ϵ* for inliers, with 16 devices and a subset size of 10 nodes.

The bounds that distinguish outliers from inliers are determined by adding, or subtracting, a threshold value *ϵ*. The value of *ϵ* should not be too small, otherwise there will be too few inliers to determine an accurate mean value. The value for *ϵ* has been determined experimentally; see Figure 5.

**Figure 6:**
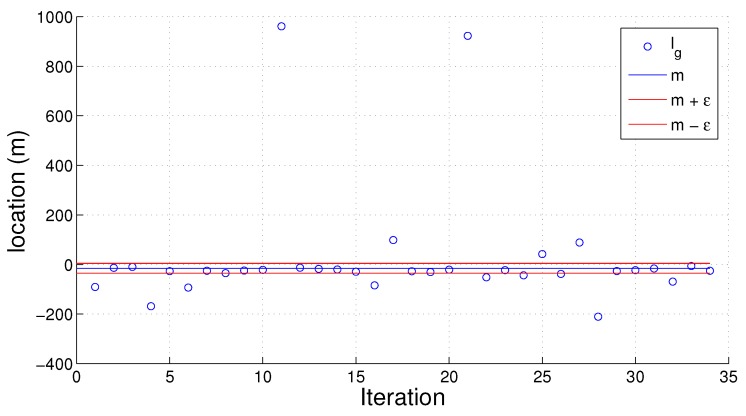
x-coordinates $\vec{x_{i}}$ for device *i*, with mean *ω* and bounds *ϵ*. ϵ=20.

An example of the set $\vec{g}$ is shown in Figure 6 as a scatter plot. The mean value *ω* and the inlier bounds *ϵ* are graphed in blue and red, respectively. All values in the area between the red lines are perceived as inliers.

It is possible that two bins receive a similar amount of counts; this means that $\vec{g}$ contains arbitrary data. The algorithm therefore uses the mean value of the centers that have either the maximum number of counts or are within 10% of the maximum count. By doing so, the most optimal value for *ω* is found.

### 4.3. Starting Point Levenberg–Marquardt Solver

The Levenberg–Marquardt (LM) solver requires an initial guess or “starting point” to start searching for an optimal solution. The solver updates each parameter so that the residual of the cost function in the next iteration is less than in the current iteration. The solver will always find a local optimum as it only converges in a “downward” direction from its starting point. The initial starting point is therefore important in order to find a local minimum that is close to the global minimum. The parameters that need to be initialized are the angles *α* towards each sound event from the center of the device constellation and the location of the devices *X*. The minimum solver proposed by Burgess et al. [20] uses linear techniques to solve the localization problem described in Section 3. The minimum solver solves the system of equations by applying matrix factorization. The matrix factorization algorithm calculates the Single Value Decomposition (SVD) of the TDOA matrix Δ. The result is used to form a symmetric matrix *C* and preforms a Cholesky decomposition [23] when *C* is positive definite. When *C* is not positive definite, the minimum solver uses a complex factorization method instead and yields a complex solution, which can still be used as an initial starting point by using the real part of the solution. While it is fast, it does not return the most optimal solution for our case; however, the result is good enough to use as an initial guess. Therefore, the minimal solver is implemented in our algorithm to obtain the initial parameter estimation.

### 4.4. Main Loop

The CLASS algorithm draws a random subset $X^{\prime }\subset X$ of *m* devices over multiple iterations. We implemented a Levenberg–Marquardt (LM) solver to minimize Equation (6) for $X^{\prime }$ and $\vec{\alpha }$ as denoted in Equation (7). We store the solutions for all devices $X_{i}^{\prime }$ and $\alpha_{i}$ for each main loop iteration *i*. A small subset will have a higher variation in the resulting locations and thus needs more iterations to determine the inliers. A larger subset will vary less and needs fewer iterations. The number of iterations *l* to run the LM solver is computed based on the subset size *m* and the total amount of devices *M*:

(12) $$l=\left\lfloor \beta \frac{M}{m}\right\rceil$$

where ⌊⌉ is the nearest integer and *β* the loop factor. *β* is the minimum number of iterations to enable robust inlier detection when m=M. The optimal subset size was determined experimentally by trying different sizes and comparing the results; these are presented in Section 5.3.

### 4.5. Outlier Detection in the Results

Because we run the main loop for *l* iterations, we acquire a set of location data for each device. This set of location data contains outliers. Outliers in the localization results are caused by the technological limitations of the hardware platform, synchronization errors and violation of the Far Field Approximation (FFA) due to sound sources that originate from near the device constellation. Sound events that originate from near or inside the constellation of devices will be invalidated by this part of the CLASS algorithm. The outliers are detected and removed by the HBOS algorithm described in Section 4.2. The remaining inliers are averaged to acquire the respective positions of all devices in *X*.

### 4.6. Complexity

In order to determine the complexity of the CLASS algorithm, we look at the individual components of the algorithm. *M* denotes the total number of devices in the constellation, *m* the number of devices in a subset and *N* the number of sound events in the dataset.

The complexity of TDOA filtering and averaging (Algorithm 1) is:

(13) $$O\left(2M^{2}N\right)=O\left(M^{2}N\right)$$

The complexity to find the initial guess is:

(14) O(mN)

The cost function J(X,α) described in Equation (6) has complexity:

(15) $$O\left(\left(m-1\right)Nm/2\right)=O\left(m^{2}N\right)$$

The complexity of the main loop is determined by the number of iterations for the main loop *l*, the number of iterations of the LM solver and the complexity of the cost function. In the best case scenario, the solver requires a single step to reach a local minimum. In this case, we assume the worst case scenario and use the maximum iterations of the LM solver, denoted as *g*. The solver updates the solution based on the derivative of the cost function. When we plug in the cost function in the solver, the main loop has complexity:

(16) $$O(lm^{2}Ng)$$

The HBOS stage of the algorithm has complexity:

(17) O(blM)

where *b* is the number of bins used by the algorithm. Combining the sub-complexities yields the overall CLASS Algorithm complexity:

(18) $$O(\left(M^{2}N\right)+\left(mN\right)+\left(lm^{2}Ng\right)+\left(blM\right))$$

Because $\left(mN\right)<\left(blM\right)<<\left(M^{2}N\right)<<\left(lm^{2}Ng\right)$, the complexity becomes:

(19) $$O(lm^{2}Ng)$$

In order for an algorithm to be usable on a smart phone, its complexity should not be too large. Modern smart phones utilize a multi-core processor with a clock frequency in the range of 2–2.5 GHz. When we fill in Equation (19), we can estimate the runtime of the CLASS algorithm. In this example, we assume the4 worst case scenario where the LM solver requires a maximum number of iterations g=6000. The number of main loop iterations l=56; the subset size m=10; and number of sound events N=20. Assuming that a CPU architecture includes a single operation floating point unit and has a processor speed of 2.5 GHz, localizing 16 devices with 20 sound events requires approximately $\left(56\times 6000\times 10^{2}\times 20\right)/\left(2.5\times 10^{9}\right)=269$ ms. Hence, taking into account any additional algorithm overhead, it is safe to say that the CLASS algorithm is able to localize a set of devices within a second. This is fast enough for most localization applications on smart phones. We also want to point out that the algorithm can easily be distributed by distributing subsets over multiple devices and aggregating the localization results in a central location. Distributing the subsets in the CLASS algorithm will reduce its complexity to:

(20) $$O(m^{2}Ng)$$

## 5. Experimental Validation

To validate the CLASS algorithm we developed an Android application that was used on multiple devices in an outdoor experiment. The application connects a group of devices and allows them to respond to sound events. In this section, we discuss the key elements of the application and present the experimental results.

### 5.1. Android Application

The following components are required for collaborative localization on mobile devices:

- Time synchronization amongst devices in the network
- Detecting sound events and recording their Time Of Arrival (TOA)
- Sharing and aggregation of time stamps
- Executing the localization algorithm with TOA data

#### 5.1.1. Time Synchronization

**Figure 7:**
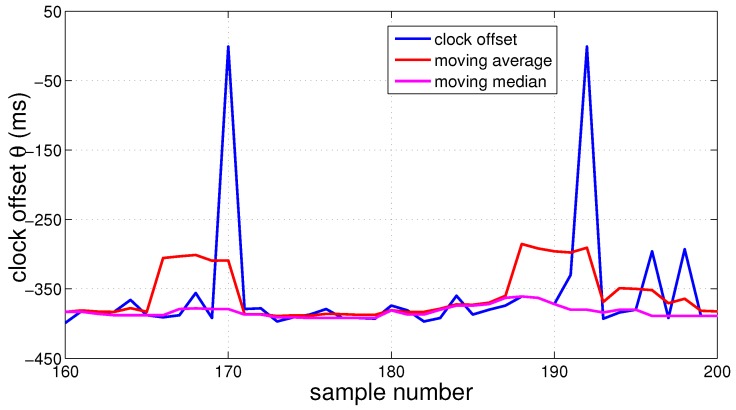
NTP clock offset, moving mean and median filtered signals. Window-size = 5 samples.

In order to obtain the location of devices and sound events, each participating device must record a time stamp as soon as it detects a distinct sound event in the environment. It is mandatory that the system clocks between the devices are synchronized. An error of 10 ms in the time stamp relates to a spatial error of 3.40 m; an accurate time synchronization smaller than 1 ms is therefore desirable. Even when Android devices are synchronized through the NTP, the error in time synchronization is significantly larger than 1 ms due to OS overhead. We used the inaccurate NTP for synchronization between the devices. The offset *θ* between the system clock and the network clock varies over multiple samples and contain outliers; see Figure 7.

**Figure 8:**
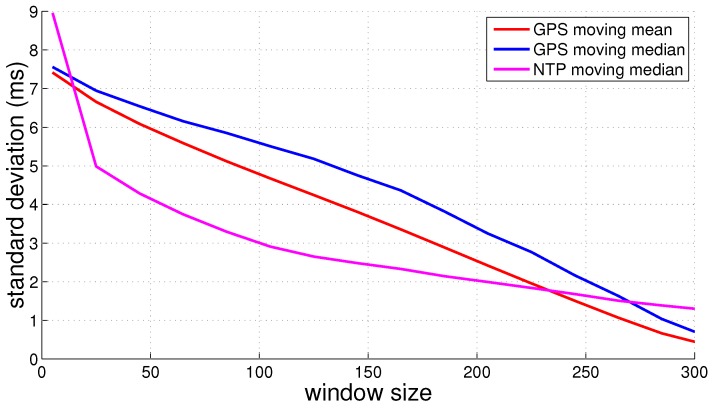
Standard deviation of the clock offset, varying the window size for GPS and NTP.

Therefore, it is required to apply a moving mean on NTP values. As denoted in Figure 8, applying a moving mean with a window of 200 samples results in a standard deviation of 2 ms in the system clock offset. During our experiment, we utilized a laptop as the NTP server. In a practical scenario, one device in the collaborative network can act as a Wi-Fi hotspot and NTP server.

For comparison, we also investigated GPS time synchronization on Android devices. Accurate time information is communicated by the GPS chip through NMEA messages and has a resolution of nanoseconds. NMEA messages are communicated to the Android framework through the GPS driver and geolocation engine. This communication overhead introduces an offset between the time inside the NMEA message and the time the NMEA message is received in the Android framework. This offset also varies and introduces jitter in the offset. The user application that handles the received NMEA message in a callback also suffers from latency. The user application handles the callback after a small delay, as Android is not a real-time OS. The Android framework provides a time stamp *λ* along with the received NMEA message. This time stamp is the device’s system time at the moment the Android framework received the NMEA message from the GLengine. We calculated the offset between the provided time stamp *λ* in the callback and the GPS time. This offset is not stable and also varies over time. Figure 8 denotes the standard deviation in system clock offset versus GPS and NTP time with an increasing moving filter window size. The difference in accuracy between utilizing GPS and NTP time synchronization is not very big. Improving the time synchronization method will reduce the number of outliers in TDOA measurements.

#### 5.1.2. Sound Event Detection

In our experimental setup, we used the sound of an air horn as a distinguished sound event. For simplicity, we used a Signal to Noise Ratio (SNR) threshold value as the sound event. The Android application records a sound event when the energy signal in the frequency band of the air horn exceeds the threshold value *λ*. In general, any sound event that has a sufficient differentiating characteristic can be used. In order to make sound-localization more practical, arbitrary sound events from the environment should be identified by their characteristics. Sound classification is an active field of study [24,25,26] and outside the scope of this work.

Most Android devices contain at least one microphone, which can be used to record audio. Android suffers from input latency, which introduces large errors in the TOA measurements [27,28]. Android needs to be universal, and therefore, high level APIs are used to support hundreds of thousands of different applications. Each manufacturer implements its individual audio hardware components. Due to the overhead that occurs between the user application and the audio hardware, significant audio latencies are introduced. This latency is not constant and varies over time. The jitter of input latency causes unpredictable and unacceptable errors in ubiquitous applications that require accurate temporal information in relation to audio information. Through measurements on multiple types of devices, we found that Android devices suffer from input latency, which can vary from approximately 50 ms–150 ms, with a worst case jitter of 300 ms.

The input latency varies with different types of hardware, but there is always jitter. We could not find a strong correlation between resource usage and input latency. Thus, the impact of user behavior on accuracy is minimal and can be excluded by implementing high priority threads for the cooperative localization application.

The first cause of latency is the analog circuitry of the device. In general, it is not possible for an end user to optimize the analog circuitry of Android devices. According to Google engineers, the analog circuitry does however not always contribute to audio latency significantly [29]. The major surface-level contributors to audio latency in Android are the following [29]:

- Application
- Total number of buffers in the pipeline
- Size of each buffer, in frames
- Additional latency after the app processor, such as from a digital signal processor

Audio buffers are usually increased or decreased to overcome buffer under and over-runs. In Google’s experience, the most common causes of under and over-runs include:

- The Linux Completely Fair Scheduler
- High-priority threads with FIFO scheduling
- Priority inversion
- Long scheduling latency
- Long-running interrupt handlers
- Long interrupt disable time
- Power management
- Security kernels

It is hard to overcome the input latency of Android without adapting its architecture. The resulting error in TDOA values will have to be dealt with in the localization algorithm.

In order to distinguish sound events from environmental noise, such as wind blowing in the microphone, the audio input is filtered. Since sound events can be distinguished by their frequency in a certain region, the input is filtered with a band-pass filter. Noise in other frequency ranges is filtered out, and the number of false triggers in sound event detection is decreased. A Fast Fourier Transformation (FFT) filter requires more calculation steps than a Finite Impulse Response (FIR) filter but is more flexible in its configuration. Because transforming the input signal to the frequency domain opens up the ability to detect audio-events by their frequency signature and its flexibility, we implemented an FFT filter in the experimental application. Implementing Digital Signal Processing (DSP), such as an FFT, filter adds a little offset in the time stamp. This can be accounted for by buffering time stamps and should not contribute significantly to the error in TDOA measurements.

After filtering out all unwanted frequencies, the energy signal in the leftover frequency band is checked against a threshold *λ*. When the signal exceeds the threshold, a time stamp (TOA) is recorded. In order to acquire a time stamp, the system clock, which is located in the Linux kernel, must be accessed from the Android Application layer. This can add additional offset in the time stamp due to OS overhead. In order to process the input data without overhead found in the Java environment, such as the memory garbage collector [30], the overhead-critical parts of our application have been developed in the native layers of Android using the Native Development Kit (NDK). The NDK is a tool set that allows developers to implement parts of an app using native-code languages, such as C and C++, in the native layer. The Open Sound library (Open-SL) [31] functions directly on top of the Android Hardware Abstraction Layer (HAL) and was implemented to achieve lower latency audio.

A difference in microphone gain can cause one device to detect a sound event earlier or later than other devices, which will cause an offset in the respective TDOA measurement for those devices. In the experimental application, this is accounted for by using a variable threshold that is depended on the microphone gain of each respective device and utilizing sound events with short onset times. Other techniques, such as the cross correlation of sound events in the frequency spectrum, will probably not suffer from this problem and can be investigated in future work.

#### 5.1.3. Sharing and Aggregation of Time Stamps

In order to aggregate the time stamps and disperse other information between devices, a network of some sort is required. In the experimental setup, we utilized a Wi-Fi-router. When a group of devices is used and no Wi-Fi network is available, it is possible to enable a mobile hotspot on one device in the network. Another possibility is to implement an ad hoc network model, such as BLESSED [32]. BLESSED is a data dissemination model for smart phones that requires no existing infrastructure. An approach such as BLESSED can be integrated with the work in this paper.

### 5.2. Outdoor Experiment

**Figure 9:**
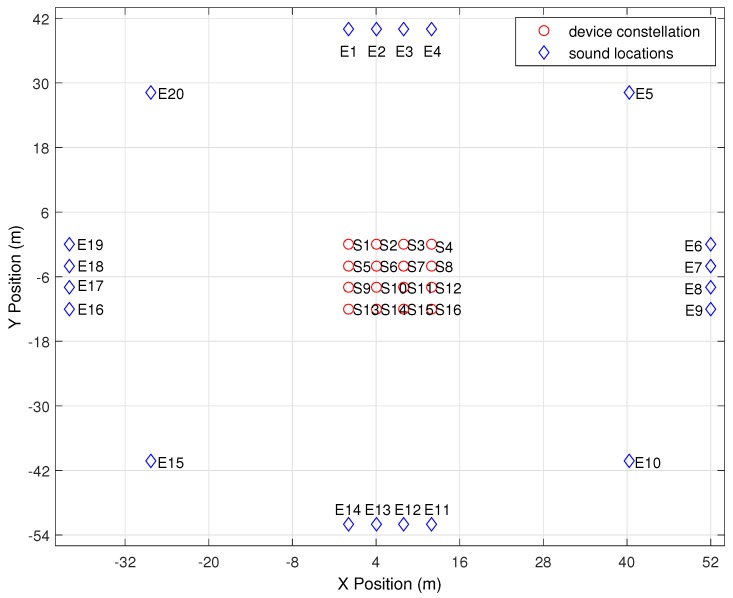
Experimental outdoor setup.

In order to obtain a realistic dataset, the main experiments took place in an outdoor environment, where the setup was exposed to wind and environmental noise. We utilized sixteen Nexus-7 (2012 edition) tablets to obtain the data that were used in this research. Figure 9 displays a top-down 2D overview of the ground truth for device locations *S* and sound event origins *E*. The devices were placed on top of tripods, at a height of approximately one meter, in a 12 m × 12 m grid with a perpendicular inter-device distance of four meters. The phones do not have to be arranged in a regular grid; the pattern used was chosen for ease of positioning and validation. The only requirement that our approach has is that the inter-device distance should not become too large when sound events originate from near the device constellation. The average inter-device distance should be around a fourth of the distance to the sound event’s origin [9,20]. Because the Android architecture already introduces very large errors in the TDOA measurements, we decided to not include samples that violate the Far Field Approximation (FFA). Different source-to-device distances have been evaluated in [9,20]. The sound events were generated forty meters away from the edge of the device constellation. Sound-events were generated from twenty different locations distributed along a circle around the device constellation. At each location, four sound events were generated. All devices were executing the application described in Section 5.1. Sound events were generated by means of an air horn, which generated a loud, distinctive sound with a certain frequency. In general, any type of sound can be used when it is loud enough and can be distinguished by all devices.

The following steps were taken during the experimental measurements. For simplicity reasons, the network was configured as a star-network. A laptop acted as the server node in the local network, and all other devices acted as a “client”. Note that sound event detection has been widely studied in the research community, and this work focuses on localization accuracy. Without loss of generality, in our experiment, we used an air horn to generate sound events. The Android application on each device was configured with a band-pass filter that filtered out environmental sounds and let the frequency band of the air horn pass through. The devices triggered when the SNR of the air horn exceeded a dynamic threshold value, as discussed in Section 5.1.2. The SNR threshold together with the location of the sound event were configured on the server device before each measurement. When a new measurement was initiated, the server device broadcast a start message, along with a measurement identification number (ID), the SNR threshold and sound event location. All devices started with a “calibration” phase. During this phase, all devices determine the noise level of the environment for three seconds. After this phase, the devices started to listen for the air horn’s signal by filtering out other sounds in the frequency domain. As soon as the energy level in the air horn’s signal reached the threshold, a time stamp was recorded on each device. A sound event was generated by sounding the horn towards the device constellation. The devices then recorded and sent a time stamp along with the measurement ID to the server. The server stored all of the values in a Structured Query Language (SQL) database. All devices connected to the server through Wi-Fi and transmitted data by the Hypertext Transfer Protocol (HTTP) POST request method. The CLASS algorithm was later executed offline with MATLAB.

### 5.3. Results

The CLASS algorithm returns a solution set for the location of all devices *X* and a set of directions *α* towards all sound event origins. Each direction $\alpha_{j}$ is denoted as a vector that originates at the center of the device constellation and is oriented towards sound event $E_{j}$. Localization without an anchor device results in a solution set that is rotated and translated. The obtained solution for the position of all devices is always a relative position to other nodes. In order to determine absolute locations and directions, at least two devices must know their geographic location. We consider the case where no devices know their geographic location. In order to evaluate the quality of the solution, it is matched to the ground truth by rotation and translation. The rotation and translation that have the best fit with the ground truth are found by using [33]. The relative error of the solution is then calculated as follows:

(21) $$RMSE=\sqrt{\frac{1}{N}\sum_{i=1}^{N}{\left({\left||X_{i}-{\bar{X}}_{i}|\right|}_{2}\right)}^{2}}$$

where *N* denotes the number of devices, ${\left||\cdot |\right|}_{2}$ the $L^{2}$ norm (Euclidean distance), $X_{i}$ the ground truth location of device *i* and ${\bar{X}}_{i}$ the estimated location.

In order to evaluate the quality of the estimated set of directions, $\bar{\alpha }$ is rotated to best fit the ground truth. The relative error for the estimation of the directions is then calculated as follows:

(22) $$RMSE=\sqrt{\frac{1}{M}\sum_{j=1}^{M}{\left(\alpha_{j}-\bar{\alpha_{j}}\right)}^{2}}$$

where *M* denotes the number of sound events, $\alpha_{j}$ the ground truth and $\bar{\alpha_{j}}$ the estimated direction towards sound event *j*.

**Figure 10:**
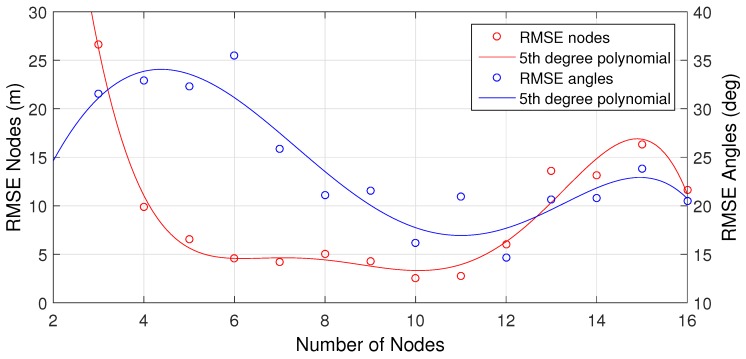
Varying the size of the subset of a constellation with a total of 16 devices.

As discussed in Section 4.4, the accuracy of the result depends on the size of the subset $X^{\prime }$. Figure 10 denotes the RMSE of the localization result for different sizes of $X^{\prime }$. The results indicate that between six and twelve devices, the number of devices does not influence the accuracy significantly. The minimum required number of devices in a subset is three; however, this will yield poor localization results. The accuracy also decreases as the number of devices in a subset approaches the same size of the total set of devices. The accuracy decreases because the LM solver will end up in similar local minima for each loop iteration *i*, and no outliers can be found in the location results. The size of subset $X^{\prime }$ during the experiments was ten.

**Figure 11:**
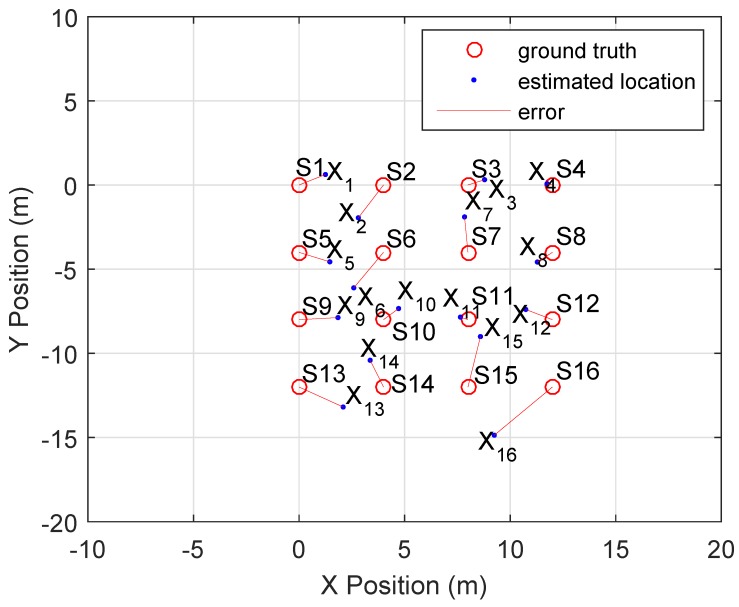
Localization results. The RMSE is 1.97 m.

The CLASS algorithm was executed ten times. A mean RMSE of 2.18 m with a standard deviation of 0.22 m is achieved. The mean RMSE of the estimated directions is 17.5° with a standard deviation of 2.3°. Figure 11 displays a top-down 2D overview of the ground truth for device locations *S*. The estimated locations *X* for each device are represented as blue dots. The RMSE for this particular solution set is 1.97 m. The localization errors are indicated by red lines, which represent the Euclidean distance.

Some devices are located further away from their ground truth than others. The errors in TDOA, for all sound events, are represented in the relative positions shown in Figure 11. An error introduced by one device will always influence the others, as TDOA values are always relative.

**Figure 12:**
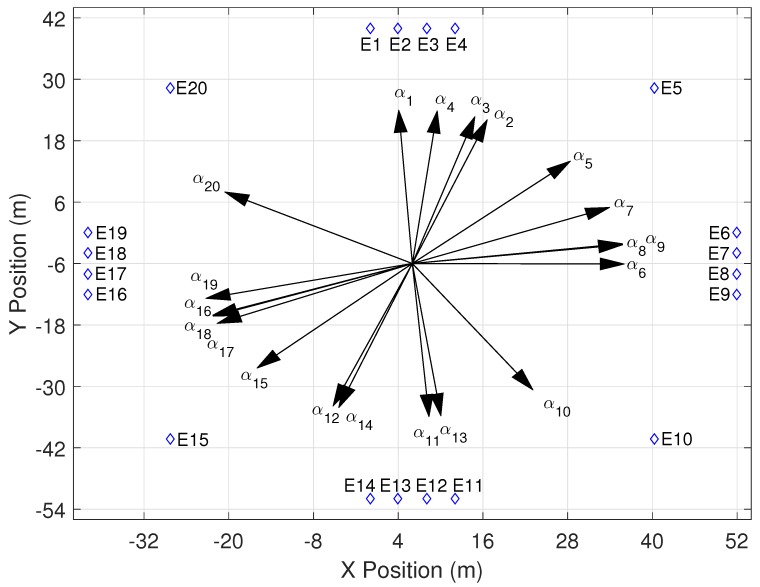
Estimation of directions towards sound event origins. The angular RMSE is 14.64°.

A solution set of the estimated directions towards the sound event origins is shown in Figure 12. The device constellation is not shown in this figure for the sake of clarity. Each direction $\alpha_{j}$ is plotted as a vector that originates at the center of the device constellation and is oriented towards sound event $E_{j}$. The angular RMSE for this particular solution set is 14.64°. The results indicate that the directions are accurate enough to use as indicators of sound event origins. An absolute position or direction must be known in order to find the true direction towards the origin of a sound event or device. This can be done by knowing the location of at least two devices and/or sound event locations. The exact location of the sound event can be triangulated with the TDOA data after the device locations have been estimated. This requires an additional step in the CLASS algorithm.

### 5.4. Comparison with Ellipsoid Method

**Figure 13:**
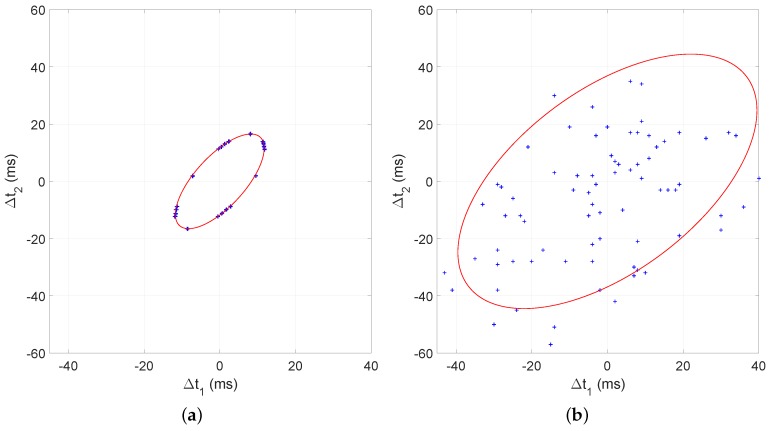
Comparison of the ellipsoids between simulated TDOA measurements and TDOA measurements obtained with our Android application. (**a**) Ellipsoid between $S_{1}$, $S_{2}$ and $S_{6}$ with simulated TDOA measurements where σ=0.1 ms; (**b**) ellipsoid between $S_{1}$, $S_{2}$ and $S_{6}$ with our real measurement data.

Compared to other works that utilize sound for localization, our results might not look impressive to some, since we use Android measurements, which contain much larger errors. Related work that utilized iPhones and laptops [10], which are significantly more deterministic, achieved an average accuracy of 38 cm, while we achieve an RMSE of 2.18 m. Accurate results can be achieved when deterministic hardware or existing infrastructure, such as anchor nodes, are used. We however do not use any anchor devices and focus on an approach that works with poor TDOA measurements obtained with indeterministic devices. In order to show how measurements are less accurate, we implemented the ellipsoid method described in [10] for comparison. TDOA measurements between three devices characterize an ellipsoid in 2D space (Figure 13). The ellipsoid equation can be used to derive the distances and angles between three devices. In Figure 13a, we plot simulated TDOA values between the positions of nodes $S_{1}$, $S_{2}$ and $S_{6}$. The devices were positioned as in our real-world experiment setup (Figure 9). A Gaussian noise with σ=0.1 ms was added to the ground truth TDOA values to represent the measurement error described by Wendeberg et al. in [10]. The simulated TDOA values are positioned on the ellipsoid (Figure 13a). The TDOA measurements derived with our Android application are plotted in Figure 13b and are not at all positioned on the ellipsoid. This is a direct result of the high variance in the TDOA measurements derived from indeterministic devices.

**Figure 14:**
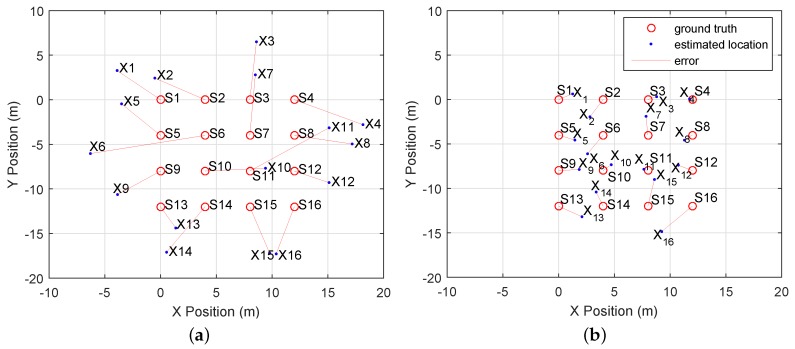
Comparison of ellipsoid [10] and CLASS localization results utilizing our real measurement data. (**a**) Ellipsoid localization results. The RMSE is 6.09 m. (**b**) CLASS localization results. The RMSE is 1.97 m.

We used our real measurement data to localize the set of devices with the ellipsoid method; the results are plotted in Figure 14a. A resulting solution set that was obtained with the CLASS algorithm is shown in Figure 14b. Over 10 experiments the ellipsoid method achieves a RMSE of 6.09 m with a standard deviation of 0.33 mm. The CLASS algorithm achieves a RMSE of 2.18 m with a standard deviation of 0.22 m. The deviation for the CLASS algorithm is higher because we utilize a different set of random subsets for each experiment, while the ellipsoid method always uses the entire set of TDOA data. The results show that the ellipsoid method is not robust enough to handle large variation in TDOA measurements. Otherwise, the CLASS algorithm shows a significant increase of localization accuracy.

## 6. Conclusions

In this paper, we have presented the CLASS algorithm. Collaborative localization on Android devices is challenging because of the technical limitations of Android. Android is not a real-time operating system and introduces large errors in Time Of Arrival (TOA) measurements. The main contributors to these inaccuracies are audio input latency and poor time synchronization capabilities. The CLASS algorithm deals with inaccurate measurements by exploiting multiple TDOA measurements from a single sound event and splitting the measurements into subsets. By doing so, the algorithm can weed out the outliers from the TDOA values and localization results. The complexity of the algorithm is small enough to execute on a modern smart phone. Our results show that it is feasible to simultaneously achieve a relative positioning of both devices and sound sources with sufficient accuracy, even when using non-deterministic devices and platforms, such as Android.
